# The single-cell landscape of cystic echinococcosis in different stages provided insights into endothelial and immune cell heterogeneity

**DOI:** 10.3389/fimmu.2022.1067338

**Published:** 2022-12-08

**Authors:** Xiaofeng Jiang, Xiaofan Zhang, Nan Jiang, Yeting Sun, Teng Li, Jing Zhang, Yujuan Shen, Jianping Cao

**Affiliations:** ^1^ National Institute of Parasitic Diseases, Chinese Center for Disease Control and Prevention, (Chinese Center for Tropical Diseases Research), Key Laboratory of Parasite and Vector Biology, National Health Commission of the People’s Republic of China, World Health Organization Collaborating Centre for Tropical Diseases, Shanghai, China; ^2^ Department of Laboratory Medicine, Sun Yat-sen Memorial Hospital, Sun Yat-sen University, Guangzhou, China; ^3^ School of Global Health, Chinese Center for Tropical Diseases Research, Shanghai Jiao Tong University School of Medicine, Shanghai, China

**Keywords:** cystic echinococcosis, single-cell sequencing, immune cells, SPP1^+^ macrophages, *Echinococcus granulosus*, angiogenesis

## Abstract

**Introduction:**

Hydatid cysts and angiogenesis are the key characteristics of cystic echinococcosis, with immune cells and endothelial cells mediating essential roles in disease progression. Recent single-cell analysis studies demonstrated immune cell infiltration after *Echinococcus granulosus* infection, highlighting the diagnostic and therapeutic potential of targeting certain cell types in the lesion microenvironment. However, more detailed immune mechanisms during different periods of *E. granulosus* infection were not elucidated.

**Methods:**

Herein, we characterized immune and endothelial cells from the liver samples of mice in different stages by single-cell RNA sequencing.

**Results:**

We profiled the transcriptomes of 45,199 cells from the liver samples of mice at 1, 3, and 6 months after infection (two replicates) and uninfected wild-type mice. The cells were categorized into 26 clusters with four distinct cell types: natural killer (NK)/T cells, B cells, myeloid cells, and endothelial cells. An SPP1^+^ macrophage subset with immunosuppressive and pro-angiogenic functions was identified in the late infection stage. Single-cell regulatory network inference and clustering (SCENIC) analysis suggested that Cebpe, Runx3, and Rora were the key regulators of the SPP1^+^ macrophages. Cell communication analysis revealed that the SPP1^+^ macrophages interacted with endothelial cells and had pro-angiogenic functions. There was an obvious communicative relationship between SPP1^+^ macrophages and endothelial cells *via* Vegfa–Vegfr1/Vegfr2, and SPP1^+^ macrophages interacted with other immune cells *via* specific ligand–receptor pairs, which might have contributed to their immunosuppressive function.

**Discussion:**

Our comprehensive exploration of the cystic echinococcosis ecosystem and the first discovery of SPP1^+^ macrophages with infection period specificity provide deeper insights into angiogenesis and the immune evasion mechanisms associated with later stages of infection.

## Introduction

Cystic echinococcosis (CE) is a severe chronic disease caused by the larval stage of *Echinococcus granulosus* and characterized by hydatid cysts in target organs (mainly in the liver and lungs) filled with hydatid cyst fluid and protoscoleces (PSCs) (1). The anti-host immune response of *E. granulosus* is *via* the formation of hydatid cysts and immunomodulation. The hydatid cysts have a tough, elastic cyst wall that swells with the infection stages, increasing pressure on organic tissue such as bile or blood vessels, which induces hepatomegaly, chronic cholestatic jaundice, and subsequently, biliary cirrhosis (2). The host can produce extremely heterogeneous immune cells to infiltrate the lesion, where their numbers, composition, and functional status are dynamic at different infection stages. *E. granulosus* has evolved sophisticated strategies to evade host immune responses and establish a long delicate balance between host protection and parasite growth (3, 4). CD68^+^ macrophages were expanded in the lesion liver samples from patients with active CE, and the M2 macrophage marker represented a dominant macrophage phenotype in patients with CE (5). The persistent infection of *E. granulosus* can span many years due to its evasion strategies. The interaction network between immune and immunosuppressive cells can increase the immune microenvironment complexity at the lesion site during infection (6, 7). Angiogenesis contributes to hydatid cyst growth, and endothelial cells are key in tissue angiogenesis (8). In an *in vitro* experiment, the cells of mice infected with late-stage *E. granulosus* promoted human umbilical vein endothelial cell (HUVEC) tube formation (9). However, the involvement of various immune cells in different *E. granulosus* infection stages in the immune response versus immunosuppression at the lesion site, and endothelial cell involvement in pro-angiogenesis, are not well understood. Therefore, the immune and immunosuppressive, and angiogenesis-related issues in parasite–host interactions require clarification.

Single-cell RNA sequencing (scRNA-Seq) is a powerful tool for detecting expression spectra to elucidate different cell roles and trace cell development. In particular, scRNA-Seq aids the identification of genetic alterations in immune cells, which presents a new strategy for tumor immunotherapy (10, 11). Single-cell analyses have become universally applied in parasite and host-cell interaction following infection. Single-cell analysis has been used to study host innate and adaptive immune responses after infection (12, 13). Recently, scRNA-Seq revealed the transcriptional heterogeneity of infiltrating immune cells in the human CE lesion microenvironment, highlighting the diagnostic and therapeutic potential of targeting certain cell types in the lesion microenvironment (14). However, more detailed immune mechanisms during different infection periods were not elucidated.

Here, we analyzed the liver tissue samples from different *E. granulosus* infection stages using scRNA-Seq. We characterized the dynamics of immune and endothelial cells during each infection phase and identified a macrophage population that emerged specifically during the later phase of infection, which were termed SPP1^+^ macrophages. The SPP1^+^ macrophages were similar to the tumor-associated macrophage (TAM) population in the immune microenvironment, which aids tumor cell immune escape and promotes tumor angiogenesis (15, 16). We determined that the SPP1^+^ macrophages overexpressed many immunosuppressive and proangiogenic genes. Monocle2 analysis revealed that the SPP1^+^ macrophages might be a class of cells in an intermediate state during monocyte differentiation into conventional macrophages.

Moreover, single-cell regulatory network inference and clustering (Scenic) analysis results suggested that *Cebpe*, *Runx3*, and *Rora* might be the key regulators through which SPP1^+^ macrophages obtain their specific functions. Our study provides the first comprehensive resolution of an immune landscape in mouse liver during different *E. granulosus* infection periods and highlights the important role of SPP1^+^ macrophages in the later stages of infection, which provides targets for immunotherapy.

## Materials and methods

### Parasites and modeling

PSCs were collected from the livers of naturally infected sheep slaughtered in the Xinjiang Uygur Autonomous Region, China. All PSCs were washed five times using normal saline containing 200 U/ml penicillin and 200 μg/ml streptomycin. PSC vitality was assessed by trypan blue exclusion testing, where the acceptable proportion of viable PSCs must reach 90%. Female BALB/c mice (aged 6–8 weeks) were purchased from Jihui Laboratory Animal Co. Ltd. Twenty-four BALB/c mice were intrahepatically inoculated with 200 μl sterile suspension containing 200 living PSCs in 0.9% NaCl solution (infected mice, n = 6 per group), and the controls (WT, n = 6) were inoculated only with 200 μl 0.9% NaCl solution. The mice were raised, housed, and treated with standard conditions and killed at 1, 3, and 6 months post-infection.

### Preparation of single-cell suspensions and magnetic-activated cell sorting

Every single-cell sample was isolated from pathological tissues, cut into small pieces (<1 mm diameter), and incubated with 2 ml collagenase II and trypsin for 1 h on a 37°C shaker. Subsequently, 4 ml Dulbecco’s modified Eagle’s medium (DMEM) was added to dilute the suspension, and the remaining large particles were removed with a 40-μm cell mesh. After 5-min centrifugation at 300 × g, the supernatant was discarded, and the cells were washed twice with phosphate-buffered saline (PBS). The cell pellet was resuspended in 1 mL red blood cell lysis buffer at 4°C for 10 min. Next, 10 ml PBS was added to the tube and centrifuged at 300 ×g for 10 min. After discarding the supernatant, the cells were resuspended in 1 mL cold calcium- and magnesium-free PBS containing 0.05% BSA. The CD45^+^ cells were purified using a magnetic cell sorting system (Miltenyi Biotec) according to the manufacturer’s instructions. In addition, we enriched endothelial cells with CD31 magnetic beads and mixed the endothelial cells and immune cells in a ratio of 1:3. Live cells were enriched with a Dead Cell Removal Kit (Miltenyi Biotec) and quantified with trypan blue.

### Single-cell RNA sequencing

According to the manufacturer’s instructions, the scRNA-Seq libraries were constructed using a Chromium Single Cell 3′ Reagent Kit version 2 (10× Genomics). Single-cell suspensions were loaded onto the Chromium Single Cell Controller Instrument to generate single-cell gel beads in emulsions (GEMs). After the GEM cells were lysed, reverse transcription reactions using barcoded full-length complementary DNA (cDNA) were performed, and the cDNA was amplified using PCR with the appropriate cycles. The amplified cDNA was fragmented, end-repaired, A-tailed, index adaptor-ligated, and library-amplified. The constructed libraries were sequenced on the Illumina NovaSeq 6000 system.

### scRNA-seq data processing

The Cell Ranger pipeline (version 5.0.0, 10×Genomics) was used to demultiplex cellular barcodes, map reads to the genome and transcriptome using the STAR aligner, and down-sample reads as required to generate normalized aggregate data across samples, producing a matrix of gene counts versus cells. We processed the UMI count matrix using the R Seurat package (version 3.1.1) (17). To remove low-quality cells and likely multiplet captures, a major concern in microdroplet-based experiments, we applied criteria to filter out cells with gene numbers < 200, UMI < 1000, or log10GenesPerUMI < 0.7. Following visual inspection of the cellular distribution of mitochondrial genes expressed, we discarded low-quality cells where >15% of the counts belonged to mitochondrial genes. Additionally, we identified doublets with the DoubletFinder package (version 2.0.2) (18). After applying these quality control criteria, 37,566 single cells were included in the downstream analyses. The library size was normalized with the Seurat NormalizeData function (17) to obtain the normalized count. Specifically, the global-scaling normalization method “LogNormalize” normalized the gene expression measurements for each cell by the total expression, multiplied by a scaling factor (10,000 by default), and the results were log-transformed. The most variable genes were selected using the Seurat FindVariableGenes function (mean.function = FastExpMean, dispersion.function = FastLogVMR) (17). The mutual nearest neighbors (MNN) by Haghverdi et al. (19) was performed to remove the batch effects in scRNA-Seq data. Graph-based clustering was performed to cluster cells according to their gene expression profile using the FindClusters function. The marker genes of each cluster were identified using the Seurat FindAllMarkers function (test.use = bimod) (1). FindAllMarkers identified positive markers for a given cluster compared with all other cells.

Differentially expressed genes (DEGs) were identified using the Seurat FindMarkers function (test.use = MAST) (17). *P* < 0.05 and |log_2_ fold-change > 0.58 were set as the threshold for significantly differential expression (SDE). GO enrichment and KEGG pathway enrichment analyses of the DEGs were performed using R based on the hypergeometric distribution.

The sequencing and bioinformatics analysis were performed by OE Biotech Co., Ltd.

### GSVA

To perform the GSVA, the GSEABase package (version 1.44.0) was used to load the gene set file downloaded and processed from the KEGG database (https://www.kegg.jp/). To assign pathway activity estimates to individual cells, we applied GSVA (20) using standard settings implemented in the GSVA package (version 1.30.0). The differences in pathway activities scored per cell were calculated with the limma package (version 3.38.3).

### Pseudo-time analysis

The developmental pseudo-time was determined with the Monocle2 package (21). The raw count was first converted from the Seurat object into the CellDataSet object with the import CDS function in Monocle. The differential Gene Test function of the Monocle2 package was used to select ordering genes (qval < 0.01) that were likely to be informative in ordering cells along the pseudo-time trajectory. The dimensional reduction clustering analysis was performed with the reduce Dimension function, followed by trajectory inference with the order cells function using default parameters. Gene expression was plotted with the plot genes in pseudo-time function to track changes over pseudo-time.

### SCENIC analysis

The SCENIC analysis was run using the motifs database for RcisTarget and GRNBoost (SCENIC version 1.1.2.2 (22), which corresponds to RcisTarget 1.2.1 and AUCell 1.4.1) with the default parameters. In detail, we identified TF binding motifs over-represented on a gene list with the RcisTarget package. The activity of each group of regulons in each cell was scored by the AUCell package.

To evaluate the cell type specificity of each predicted regulon, we calculated the RSS based on the Jensen-Shannon divergence (JSD), a measure of the similarity between two probability distributions. Specifically, we calculated the JSD between each vector of binary regulon activity overlapping with the assignment of the cells to a specific cell type (23). The connection specificity index (CSI) for all regulons was calculated with the scFunctions package (https://github.com/FloWuenne/scFunctions/).

### CellChat analysis

Cell–cell communication analysis was performed using the R CellChat (v 1.1.3) package (24). First, the normalized expression matrix was imported to create the CellChat object with the create CellChat function. Second, the data were preprocessed using the default parameters with the identifyOverExpressedGenes, identifyOverExpressedInteractions, and projec tData function. Then, the potential ligand–receptor interactions were calculated with the compute CommunProb, filter Communication (min.cells = 10), and compute CommunProbPathway functions. Finally, the cell communication network was aggregated using the aggregate Net function.

## Results

### scRNA-seq profiling in mouse liver ecosystem after *E. granulosus* infection

**Figure 1:**
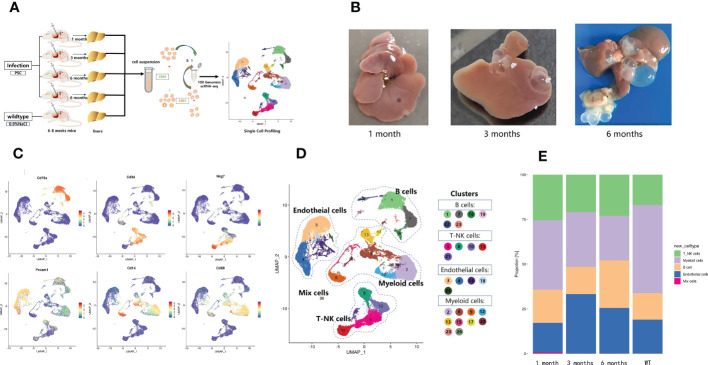
Diverse cell types in mouse liver with *Echinococcus granulosus* infection delineated by single-cell transcriptomics. **(A)** Schematic representation of the experimental strategy. scRNA-Seq was applied to immune and endothelial cells from the livers of WT mice and mice infected with *E. granulosus*; **(B)** *E. granulosus* PSCs in mouse livers at 1, 3, and 6 months post-infection; **(C)** Featureplots showing the expression levels of specific markers for each cell type. All cell types could be identified based on those classical marker genes. The gray to red color key indicates low to high relative expression levels; **(D)** The UMAP projection of 45,199 CD45^+^ liver cells from mouse liver samples shows the formation of 26 main clusters. Each point represents a single cell colored according to cluster designation; **(E)** The histogram of the proportions of cell populations identified in each infection period.

The heterogeneity of liver immune cells in mice infected with *E. granulosus* was explored using the infected mouse livers at 1, 3, and 6 months post-infection and normal saline control (wild-type, WT). The immune and endothelial cells isolated from the mouse livers were studied with snRNA-seq (**Figure 1A**). *E. granulosus* cysts began to form on the liver at 1 month post-infection, and as infection progressed over time, the cysts enlarged and became more obvious (**Figure 1B**). The immune microenvironment of the infected site changed. Afterquality control, a total of 45,199 cells were acquired for further analysis. The percentage of mitochondrial counts, nGene (normalized genes), and nUMI (normalized unique molecular identifier) distributions are shown in **Supplementary Figure S1A**. Dimensionality reduction and cluster analysis grouped the cells into 26 clusters. Each cluster was compared to the other pooled clusters to identify unique gene signatures, and the top 10 marker genes of each cluster were represented in a heatmap (**Supplementary Figure S1B**). Cluster-specific genes were used to annotate cell types with classic markers described in previous studies: B cells (*Cd79a* ^+^), T and natural killer (NK) cells (*Nkg*7^+^ and *Cd3d* ^+^), endothelial cells (*Pecam1* ^+^), and myeloid cells (*Cd68* ^+^ and *Cd14* ^+^) (**Figure 1C**). Therefore, based on the expression of the classical marker genes, we identified eight major cell types, which included B cells (cluster 1, 7, 16, 19, 22, 23), T and NK cells (cluster 5, 8, 10, 11, 21), endothelial cells (cluster 3, 4, 14, 18, 24), and myeloid cells (cluster 2, 6, 9, 12, 13, 15, 17, 20, 23, 26) (**Figure 1D**). Analysis of the number of cell types changes revealed that the relative abundance of myeloid cells decreased stepwise from WT to 6 months (**Figure 1E**). The relative abundance of endothelial cells at 3 and 6 months was higher than that in the other samples (**Figure 1E**). In addition, B cells were significantly increased at 6 months (**Figure 1E**). The findings indicated that the parasite infection changed the characteristics of liver immune infiltration and that different infection periods featured specific ecosystems.

### Dynamic changes in lymphoid cell populations and expansion of B cells expressing immunosuppressive molecules during late infection

**Figure 2:**
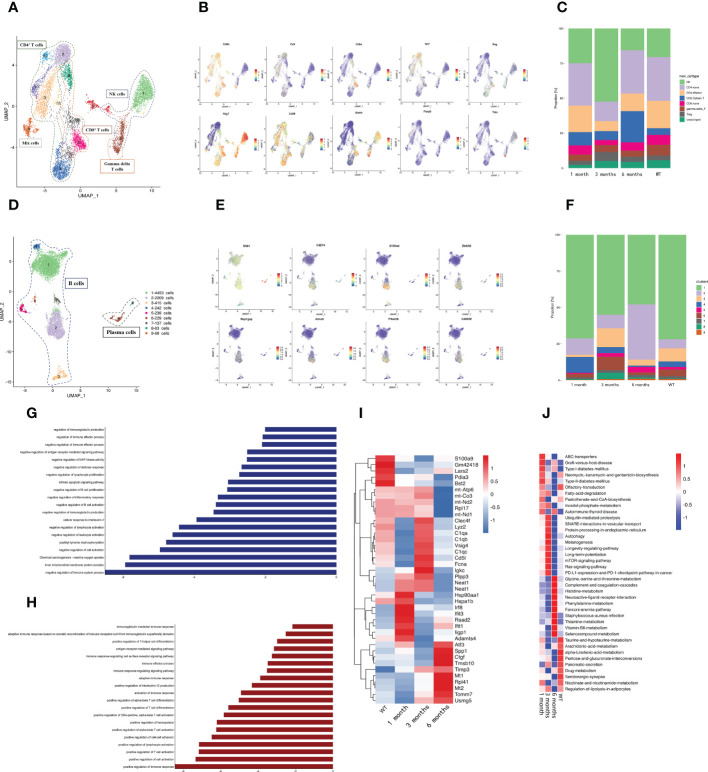
A distinct microenvironment in mouse liver after *E. granulosus* infection. **(A)** The UMAP plot displays 13 NK/T cell subclusters; **(B)** The expression levels of specific markers for each NK/T cell type are plotted onto a UMAP map; **(C)** The histogram depicts the proportions of NK/T cell subclusters in each infection period; **(D)** The UMAP plot shows the 9 B cell subclusters; **(E)** The expression levels of specific markers for each B cell type are plotted onto the UMAP map; **(F)** The histogram shows the proportions of B cell subclusters in each infection period; **(G)** The bar map shows the enrichment pathways of the Top 200 genes in B2 cells; **(H)** The bar map depicts the enrichment pathways of the Top 200 genes in B4 cells; **(I)** The heat map shows the endothelial cells’ highly differentially expressed genes identified during each infection period. Dark blue indicates lower expression; dark red indicates higher expression; **(J)** The heat map shows the difference in pathway activities scored by GSVA per endothelial cell between each infection period.

We delineated the change in lymphoid cells at different infection periods by re-clustering the clusters. The NK/T cell re-clustering revealed 13 populations, which included NK cells, three CD4^+^ T cell subtypes, two CD8^+^ T cell subtypes, and gamma-delta T cells (**Figure 2A**). Among the CD4^+^ T cells, CD4^+^ naïve T cells (cluster 2, 3, 12, 13) were identified by high *Cd28* expression and low *Ifng* expression, CD4^+^ effector T cells (cluster 4, the left part of cluster 11) were identified *via* high *Ifng* expression, and T regulatory cells (Tregs) (cluster 10) were classified by high *Foxp3* expression (**Figure 2B**). Among the CD8^+^ T cells, CD8^+^ naïve T cells were identified by high *Cd28* expression and low *Ifng* expression, and cytotoxic T cells were identified by high *Nkg7* expression and *Gzmb* expression (**Figure 2B**). The cytotoxic T cells underwent expansion 6 months post-infection (**Figure 2C**), which implied that CD8^+^ cytotoxic T cells were important in the host anti-*E. granulosus* immune response during this period. Gamma delta T cells were characterized by high *Trdc* expression no *Cd4* and or *Cd8a* expression (**Figure 2B**). The NK subsets (cluster 1, the upper part of cluster 6, the right of cluster 11) expressed the classical signature gene *Nkg7* and no *Cd3d* (**Figure 2B**). Compared with the other samples, NK cells were enriched at 3 months post-infection (**Figure 2C**). We speculated that NK cells were important for eliminating parasites during this period.

Re-clustering the B cells revealed that cluster 1, 2, 4, 5, 7, and 9 were classical B cells, and cluster 6 and 8 were plasma cells with *Mzb1* expression (**Figures 2D, E**). The proportion of cluster 2 changed significantly at different infection times, increasing significantly at 6 months post-infection, whereas the proportion of cluster 4 gradually decreased with the infection course (**Figure 2F**). Furthermore, we detected a characteristic expression profile in cluster 4, which had higher *Cd274*, *S100a4*, *Zbtb32*, *Cd300lf*, *Prkar2b*, *Rap1gap*, and *Armc3* expression than the other subclusters (**Supplementary Figure S2** **) (** **Figure 2E**). *Cd274* and *Zbtb32* negatively regulate immunity (25–27), and *S100a4*, *Rap1gap*, *Armc3*, and *Prkar2b* promote tumor metastasis (28–30), This was consistent with the emergence of immunosuppression in the later stages of *E. granulosus* infection and with the hydatid cyst metastasis. Gene ontology (GO) and Kyoto Encyclopedia of Genes and Genomes (KEGG) enrichment analysis revealed that cluster 2 exhibited a preference for genes involved in the following pathways: negative regulation of the immune system process and negative regulation of immunoglobulin production (**Figure 2G**) and cluster 4 exhibited strongly contributed to promoting the immune response and inflammation (**Figure 2H** **),** suggesting that the increased cluster 2 and decreased cluster 4 at 6 months post-infection may collectively contribute to the immunosuppressive microenvironment of the host at later infection stages.

### Endothelial cell functions at each infection stage

Endothelial cells are abundant non-immune infiltrating cells that are vital conduits for nutrient and oxygen delivery, waste removal, and immune cell trafficking. The *E. multilocularis* PSC isomerase (EmPGI) stimulates endothelial cell proliferation, which may support metacestode proliferation (31). The expression heatmaps of the endothelial cell genes in the sample revealed that DCN and IFN-activated endothelial cell marker genes (*Irf8*, *Rsad2*, *Ifit1*, *Ifit3*, *Iigp1*) were significantly upregulated at 1 month post-infection as compared with the WT group (**Figure 2I**). *Fcna* and *C1q* were significantly upregulated at 3 months post-infection. *Fcna* is a collagen trimer component (32), and *C1q* induces endothelial cell adhesion and spreading, binds to cell surface receptors, and stimulates inflammation (33). The differential gene expression at the different infection stages revealed that the endothelial cells overexpressed fibrosis- and angiogenesis-related genes at the late infection stage, such as *Atf3*, *Ctgf*, *Mt1/2*, *Spp1*, *Rpl41*, *Tmsb10*, and *Tomm7* (34–39) (**Figure 2I**), among which *Ctgf* promotes fibrosis and angiogenesis in endothelial cells (40, 41). Gene set variation analysis (GSVA) revealed that graft-versus-host disease and the allograft rejection pathway were activated in the early infection period (**Figure 2J**). The RAP1, mTOR, and platelet activation pathways were activated during the infection period. cAMP–EPAC–RAP1 signaling decreases cell permeability by enhancing vascular endothelial cadherin-mediated adhesion aligned by rearranged cortical actin (42). Inflammation stimulates endothelial cells and platelets in ways that affect the immune response and hemostasis (43). The *E. multilocularis* metacestode stage produces mTOR (44), where the mTOR signaling pathway is essential for angiogenesis. The cytokine–cytokine receptor interaction pathway was significantly enriched in the late infection period and mainly influenced the hepatic metabolic capacity.

### An immunosuppression macrophage subset demonstrating time-specificity for infection

**Figure 3:**
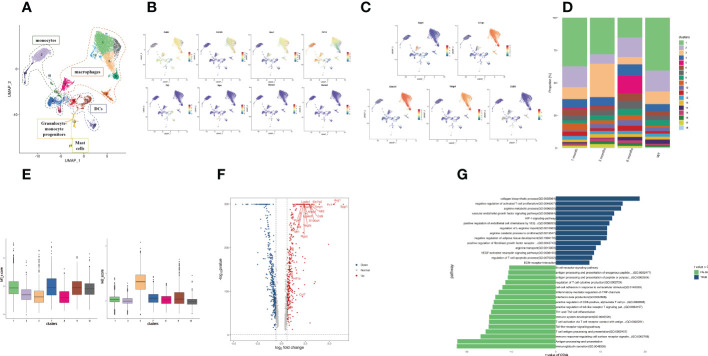
Profiling of SPP1^+^ macrophage function. **(A)** The UMAP plot displays 18 myeloid cell subclusters; **(B)** The expression levels of specific markers for each myeloid cell type are plotted onto a UMAP map; **(C)** The expression levels of specific markers for each macrophage subtype were plotted onto the UMAP map; **(D)** The histogram indicates the proportions of myeloid cell populations in each infection period; **(E)** The box plots show the classification of macrophage clusters into M1 and M2. **(F)** The volcano plot of DEGs between the SPP1^+^ and C1QC^+^ macrophages. Upregulated and downregulated genes are highlighted in blue and red, respectively; **(G)** The bar plot depicting the GSVA results of SPP1^+^ and C1QC^+^ macrophage differential genes.

The number of myeloid cells varied greatly during infection, so we performed a subcluster analysis of the myeloid cell types. A total of 18 clusters emerged within the myeloid lineage (**Figure 3A**), among which macrophages (clusters 1, 3, 5, 7, 8, 9, 12) were characterized by high *Cd163*, *Cd68*, and *Mrc1* expression (**Figure 3B**). The monocyte subsets (clusters 2, 4, 14, 15, 16) were distinguished by the classical expression of *Cd14* (**Figure 3B**). Three dendritic cell (DC) subsets (clusters 6, 10, 11) were identified by high *Flt3* expression (**Figure 3B**). Granulocyte-monocyte progenitor cells (cluster 13) were identified by high expression of *Ms4a3* and *Mpo*. Based on the high *Ms4a2* expression, cluster 17 was identified as mast cells (**Figure 3B**).

Macrophages are considered a highly heterogeneous cell population. Over the years, many studies have been conducted on TAMs, which are critical for tumor progression in tumor immune microenvironments. A proportion of TAMs is important in angiogenesis and immunosuppression (45, 46), which is related to the prognosis of patients with tumors. Here, we detected two groups of macrophages with gene signatures similar to that of TAMs. We identified these macrophages as C1QC^+^ macrophages (cluster 1, 3, 7, 8, 9, 12) and SPP1^+^ macrophages (cluster 5) *via* their high gene expression (**Figures 3A, C**). Among the C1QC^+^ macrophages, Kupffer cells (Cluster 1, 3, 7, 8, 12) were identified by high expression of Clec4f and Vsig4. Expressing the activation marker CD93, cluster 9 was identified as C1QC^+^CD93^+^ macrophages (47). We detected the expansion of SPP1^+^ macrophages in the infected mouse liver at 6 months (**Figure 3D**). The dichotomy between the C1QC^+^ macrophages and SPP1^+^ macrophages could not be fully explained based on the expression analysis of the genes related to the “classical activation” (M1) and “alternative activation” (M2) macrophages in our samples. The SPP1^+^ macrophages might be more similar to M2 macrophages and have an immunosuppressive function (**Figure 3E**). Further comparison of differentially expressed genes between myeloid cells revealed that the SPP1^+^ macrophages expressed a set of angiogenesis-related genes (*Arg1*, *Vegfa*, *Anxa1*, *Ccl7*). *Ferraro et al.* (48) demonstrated that *Anxa1* promoted *Vegfa* expression, promoting angiogenesis. The SPP1^+^ macrophages expressed fibrosis-related genes (*Fn1*, *Mt1*, *Mt2*, *Lgasl3*, *Timp1*, *Cd9*, *Trem2*). CD9^+^ TREM2^+^ macrophages play a key role in liver fibrosis (49). The SPP1^+^ macrophages expressed a set of immunosuppressive genes (*Arg1*, *Trem2*, *Pgk1*, *Lgasl1*, *Slc7a2*, *S100a4*, *Rgcc* ***)*** (**Figure 3F**). Although we cannot determine whether SPP1^+^ macrophages are M2 macrophages, high expression of *S100a4* and *Rgcc* polarizes macrophages to immunosuppressive M2 macrophages (50, 51). *Arg1* expression was enhanced in multiple myeloid cells from the peritoneum and promoted immune evasion of *E. granulosus* in mice by inhibiting the expression of the T cell receptor CD3ζ chain (52).

GSVA revealed strong enrichment of immunosuppression (arginine metabolic process, regulation of T cell apoptotic process, negative regulation of activated T cell proliferation, arginine transport), angiogenesis (VEGF-activated neuropilin signaling pathway, vascular endothelial growth factor signaling pathway, positive regulation of endothelial cell chemotaxis by VEGF, HIF-1 signaling pathway), fibrosis (negative regulation of adipose tissue development, collagen biosynthetic process, positive regulation of fibroblast growth factor receptor), and ECM receptor interaction in the SPP1^+^ macrophages, and enrichment of proinflammatory response in the C1QC^+^ macrophages (**Figure 3G**). ECM receptor interaction might be related to hydatid cyst formation. These results were consistent with the angiogenesis, immunosuppression, and fibrosis that occurred in the middle and late infection stages.

### Transcription factors regulated the gene regulatory networks identifying SPP1^+^ macrophage function

**Figure 4:**
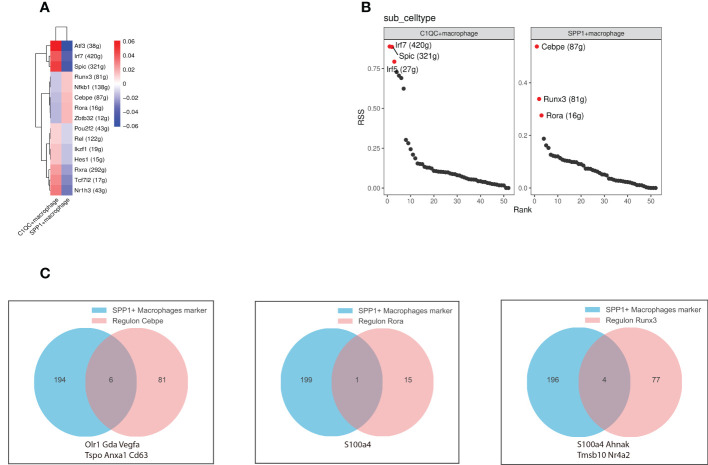
Gene regulatory networks between SPP1^+^ and C1QC^+^ macrophages. **(A)** The heatmap depicts the difference in regulon activity scores (RAS) between SPP1^+^ and C1QC^+^ macrophages; **(B)** The scatter plots show the regulons ranked based on the regulon specificity score (RSS). The red dots represent the top specific regulon. **(C)** The venn diagram depicts the intersection of the downstream genes of specific regulators (left to right regulators are Cebpe, Rora, and Runx3, respectively) with the top 200 genes of SPP1^+^ macrophages. Intersecting graphs represent genes where both intersected.

TFs and their downstream regulated genes constitute a complex intermingled gene regulation network that determines and maintains cell identity. We performed SCENIC analysis to infer the activity of regulons (comprised of a TF together with its target genes) (22) of the SPP1^+^ and C1QC^+^ macrophages (**Figure 4A**). We inferred the SPP1^+^ macrophage regulons by SCENIC analysis complemented by transcriptional regulators. Based on the regulon specificity score (RSS), *Cebpe*, *Runx3*, and *Rora* were identified as the most specific regulons associated with the SPP1^+^ macrophages, while *Atf3*, *Irf7*, and *Spic* were identified as the most specific regulons associated with the C1QC^+^ macrophages (**Figure 4B**). To explore the effect of *Cebpe*, *Runx3*, and *Rora* on SPP1^+^ macrophage phenotype and function, we intersected the top 200 specific genes and their downstream genes (**Figure 4C**). *Cebpe* is a myeloid-specific TF that is a critical mediator of myelopoiesis (53) and regulates 87 downstream genes, including *Anax1* and *Vegfa* (54). As mentioned earlier, these two genes are important in promoting angiogenesis. The intersection of the downstream genes of *Runx3* and *Rora*, and the top 200 specific genes was *S100a4*. It has been well-documented that *S100a4* is critical for macrophage polarization to an immunosuppressive type (55), suggesting that *Cebpe*, *Runx3*, and *Rora* might act as core TFs in the regulation of angiogenesis immunosuppression and liver tissue 6 months post-infection.

### Pseudo-time reconstruction exploration of the origin of SPP1^+^ macrophages

**Figure 5:**
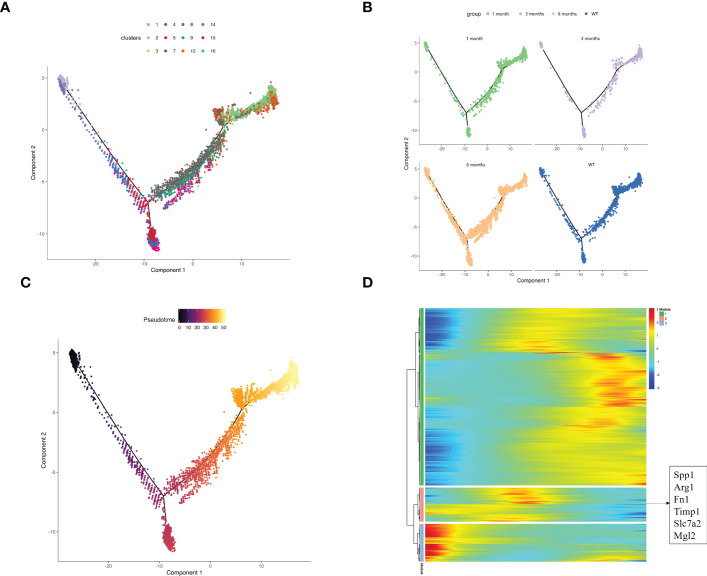
Exploring the source of SPP1^+^ macrophages based on pseudo-time analysis using Monocle2. **(A)** The pseudo-time trajectory plot represents macrophage and monocyte development. The dot color represents the cluster number; **(B)** The pseudo-time trajectory plots demonstrate the sample distribution along the trajectory. The dot color represents the group; **(C)** The developmental pseudo-time of monocytes and macrophages was inferred by Monocle analysis. The dark to bright color key indicates cell differentiation progression and the dark to bright color key progresses from the monocytes to macrophages; **(D)** The heatmap displays the SDE genes during the trajectory. The blue-to-red color key indicates low to high relative expression levels.

There are two sources of macrophages in the liver: monocyte-derived macrophages (MDMs) and tissue-resident macrophages (TRMs), which are independent of the hematopoietic system and can self-renew and be maintained in local areas (56). We performed pseudo-time reconstruction of monocytes and macrophages in the myeloid cells of liver samples to identify the origin of SPP1^+^ macrophages (**Figure 5A**). Combined with pseudo-time analysis, the leftmost monocytes began the trajectory and differentiated progressively to the right. Cluster 5 SPP1^+^ macrophages appeared in the middle of the overall trajectory, whereas the C1QC^+^ macrophage subset appeared at the end. This trajectory analysis revealed that the SPP1^+^ and C1QC^+^ macrophages were derived from monocytes and that they were intermediate cells that were ultimately converted to C1QC^+^ macrophages. We matched these cells from different samples to the trajectory and demonstrated that the number of intermediate cells increased significantly in the 6-month samples compared with other samples. These findings suggest that in the immune microenvironment of the 6-month samples, SPP1^+^ macrophages with immunosuppressive function did not continue to develop into C1QC^+^ cells with positive immune responses but stagnated in the intermediate state of immunosuppression (**Figure 5B**).

Our data demonstrated the dynamic gene expression profiles during monocyte and macrophage development (**Figures 5C, D**). The highly expressed genes in module 2 appeared in the middle of the pseudotime and were also the signature genes of SPP1^+^ macrophages, corresponding to the SPP1 macrophage location in our description trajectory.

### The SPP1^+^ macrophage subset as the core of a predicted cell–cell interaction network

**Figure 6:**
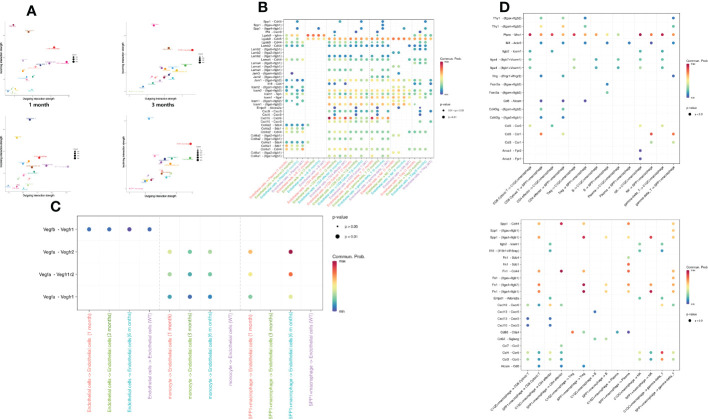
Intercellular communication of immune and endothelial cells in CE. **(A)** The bubble plot depicts interaction strength of different cell types as receptors or ligands in each sample. The outgoing (incoming) strength was gradually intensified along the abscissa (ordinate) axis. Each bubble is colored according to the cell type; **(B)** The bubble plot depicts the ligand–receptor connections involved in angiogenesis between endothelial cells and immune cells. Bubble size indicates the statistical significance and bubble color indicates the interaction level; **(C)** The bubble plot depicts ligand–receptor connections in immune cell recruitment between endothelial cells and immune cells. Bubble size indicates the statistical significance and bubble color indicates the interaction level; **(D)** The bubble plot depicts ligand–receptor connections between SPP1^+^/C1QC^+^ macrophages and other immune cells. Bubble size indicates the statistical significance and bubble color indicates the interaction level.

To characterize intercellular interactions in liver tissue after *E. granulosus* infection, we inferred cell–to cell interactions based on ligand-receptor signaling from our high-resolution scRNA-Seq data. If one cell expresses a receptor or ligand, the “ligand–receptor” interaction is defined as entering or leaving the cell. C1QC^+^ macrophages and endothelial cells had strong outgoing interactions across the five samples and SPP1^+^ macrophages had the strongest outgoing interactions in the infected mouse liver at 6 months. (**Figure 6A**).

As stromal cells, endothelial cells can recruit immune cells to the infected site. We determined that ligand pairs associated with lymphocyte recruitment varied significantly across samples. *Spp1*–integrins/*Cd44* were present only in the 6-month samples, and *Cxcl4*–*Cxcr3* and *Entpd*–*Adora2a* were present only in the 3-month samples, while *Cxcl16*–*Cxcr6* expression gradually decreased between T cells and endothelial cells as infection progressed (**Figure 6B**). We observed that the intercellular signal communication of endothelial cells became stronger with infection time. Endothelial cells are critical during angiogenesis. Notably, we found that the angiogenesis-related receptors on the endothelial cells interacted closely with the corresponding receptors on the SPP1^+^ macrophages, which were characterized by high *Vegfa*–*Vegfr1*/*Vegfr2* expression (**Figure 6C**). However, as mentioned above, the SPP1^+^ macrophages appeared only almost in the 6-month samples. This finding indicated that interaction between endothelial cells and SPP1^+^ macrophages was of great importance for angiogenesis in late-stage *E. granulosus* infection.

To explore the contribution of SPP1^+^ macrophages to the immune regulation in the 6-month samples, we compared the attraction strengths of ligand–receptor pairs from SPP1^+^ and C1QC^+^ macrophages separately for interaction with other immune cells. We not only determined that many ligand–receptor pairs existed only in the interaction of SPP1^+^ macrophages with other immune cells, among which were ligand– receptor pairs acting on SPP1^+^ macrophages, such as *Thy*–integrins, *Mif*–*Ackr3*, and *Ccl5*–*Ccr1*, but also that the SPP1^+^ macrophages acted on other immune cells, such as *Spp1*–receptors and *Fn1*–receptors (**Figure 6D**). These specific ligand–receptor pairs might be relevant for interaction SPP1^+^ macrophages with other immune cells to exert immune functions.

## Discussion

This study comprehensively characterized the heterogeneity of immune and endothelial cells in mouse liver with *E. granulosus* infection. A group of particularly amplified SPP1^+^ macrophages was present in the 6-month sample compared to the other samples, indicating well-functioning immunosuppression after *E. granulosus* infection. Analyses of the highly expressed genes and gene enrichment revealed that the SPP1^+^ macrophages were related to immunosuppression, angiogenesis, and fibrosis in late-stage *E. granulosus* infection. We speculated on the source of these cells with cell trajectory analysis and surmised *via* SCENIC analysis that *Cebpe*, *Runx3*, and *Rora* might be the key TFs for these cells to function.

Recent studies have reported macrophages highly expressing *SPP1*. In gliomas, *PTEN* deficiency activated the *YAP1* gene, which directly upregulated the expression of lysyl oxidase (LOX), a macrophage chemoattractant that activated *YAP1* in the β1 integrin–PYK2 pathway in macrophages, which in turn secreted high amounts of *SPP1*, thereby maintaining glioma cell survival and stimulating angiogenesis within the tumor (36). LOX is a downstream molecule of *HIF1α*. Here, we detected high HIFα expression, and perhaps advanced cystic–type hydatid disease shares pro-angiogenic pathways with glioma. In liver and colorectal cancers, SPP1^+^ macrophages were associated with tumor pro-angiogenesis and prognosis and were specifically present in metastatic colorectal cancer but not metastatic liver cancer (57). In colon cancer fibrosis, a positive correlation was reported between tumor–specific FAP^+^ fibroblasts and SPP^+^ macrophages in the colorectal cancer-containing cohort, and immunofluorescence staining and spatial transcriptomics verified their close localization (58). A dense fibrous capsule forms between the capsule and tissues in late- to middle-stage cystic-type hydatid disease and may be analogous to this mechanism. SPP1 specifically appeared after myocardial infarction and was almost exclusively produced by galectin-3^+^CD206^+^ macrophages. The IL-10–STAT3–galectin-3 axis was essential to generate *SPP1* for reparative macrophage polarization after myocardial infarction, and these macrophages promoted tissue repair by promoting the clearance of fibrotic and apoptotic cells (59). In lung adenocarcinoma, single-cell sequencing identified a macrophage population overexpressing *SPP1* and *VEGFA* (60).

Significant liver fibrosis was observed in liver lesion samples of patients with active cysts. Macrophages may contribute to fibrosis by producing large amounts of the profibrotic cytokines MIF and ECM1 to promote liver fibrosis in the CE lesion microenvironment (5). In our study, MIF and the ECM interactive pathway were highly expressed and enriched in the SPP1^+^ macrophages.

The immune response interaction between *E. granulosus* and the host is complex and includes the effective insecticidal immune mechanism implemented by the host. The parasites subsequently regulate these mechanisms to escape the host’s immune response (4, 61). The variability and severity of clinical manifestations of CE are related to the infection duration and intensity (62). Our study provides a basis for explaining these complex mechanisms and a reference for subsequent researchers. We will also explore the specific functions and in-depth mechanisms of SPP1^+^ macrophages further to fully elucidate the important role of this cell population during infection.

## Conclusion

Given the cellular complexity and dynamics of CE, insight into the functional contribution of each cell subtype to this disease will help to explain the CE pathogenic mechanisms and develop drugs with specific targets. Our scRNA-Seq results delineated the immune cell and endothelial cell landscape during different *E. granulosus* infection periods and revealed that SPP1^+^ macrophages are important in immunosuppression and angiogenesis during the later stages of infection. Therefore, this could be a potential therapeutic target for CE.

## Data availability statement

The raw sequence data reported in this paper have been deposited in the Genome Sequence Archive of the China National Center for Bioinformation (https://ngdc.cncb.ac.cn/gsa) under accession number CRA008416.

## Ethics statement

The animal study was reviewed and approved by the Laboratory Animal Welfare and Ethics Committee (LAWEC), National Institute of Parasitic Diseases, Chinese Center for Disease Control and Prevention, (Chinese Center for Tropical Diseases Research).

## Author contributions

XJ, XZ, and NJ conducted experiments, performed critical data analyses, and interpreted results. YSu, TL, and JC participated in the data interpreting and statistical analysis. YSh and JC designed the experiments and revised the manuscript. JZ revised the manuscript. XJ and XZ wrote the article and contributed equally to this work. All authors contributed to the article and approved the submitted version.

## Supplementary material

Supplementary Figure 1
**(A)** Distribution of the percentage of mitochondrial counts, nGene, and nUMI before (top) and after (bottom) quality control. **(B)** Heat map of the top 10 marker genes of the 26 clusters identified in all cells. The top 10 cluster-specific markers were selected based on the average log (fold change).

Supplementary Figure 2
**|** Heat map of the top 10 marker genes for the nine B cell clusters. The top 10 cluster-specific markers were selected based on the average log (fold change).
