# Supplementary figures and images for: The single-cell landscape of cystic echinococcosis in different stages provided insights into endothelial and immune cell heterogeneity
Source: Front Immunol. 2022 Dec 8;13:1067338. doi: 10.3389/fimmu.2022.1067338 (PMC9772464; doi:10.3389/fimmu.2022.1067338)

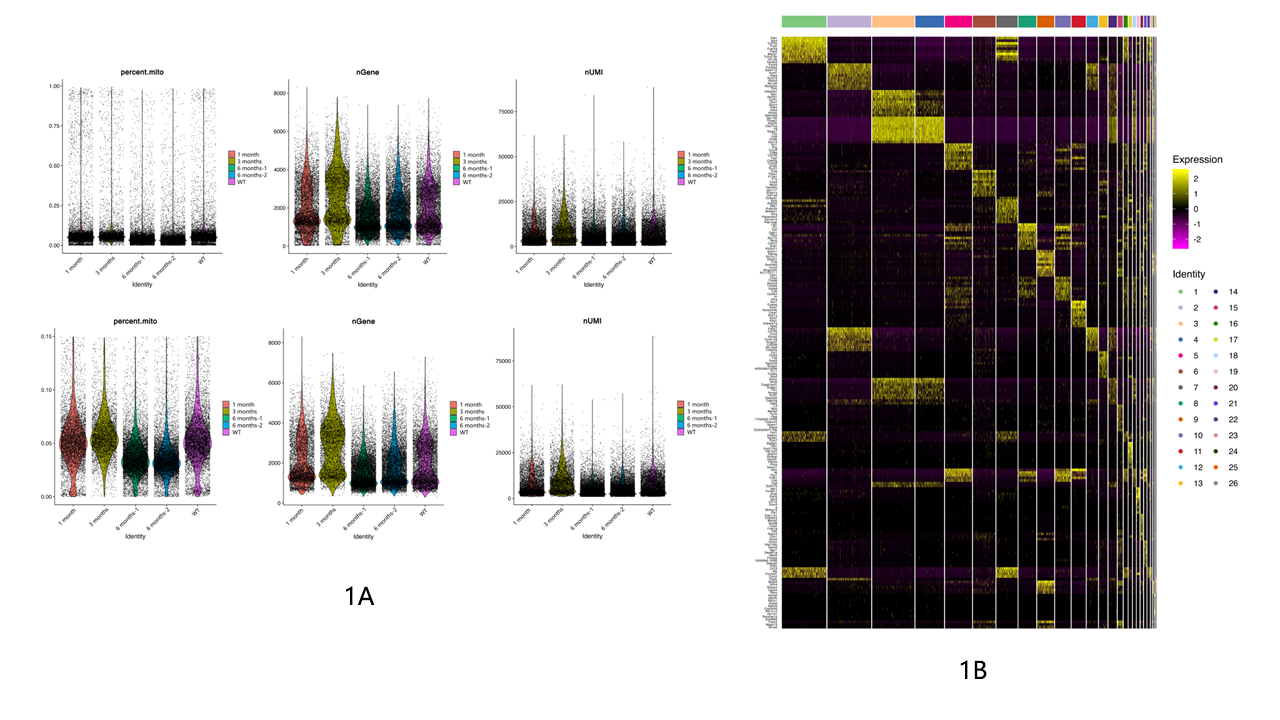

Supplement: Supplementary Figure 1 — (A) Distribution of the percentage of mitochondrial counts, nGene, and nUMI before (top) and after (bottom) quality control. (B) Heat map of the top 10 marker genes of the 26 clusters identified in all cells. The top 10 cluster-specific markers were selected based on the average log (fold change). [file Image_1.tif]

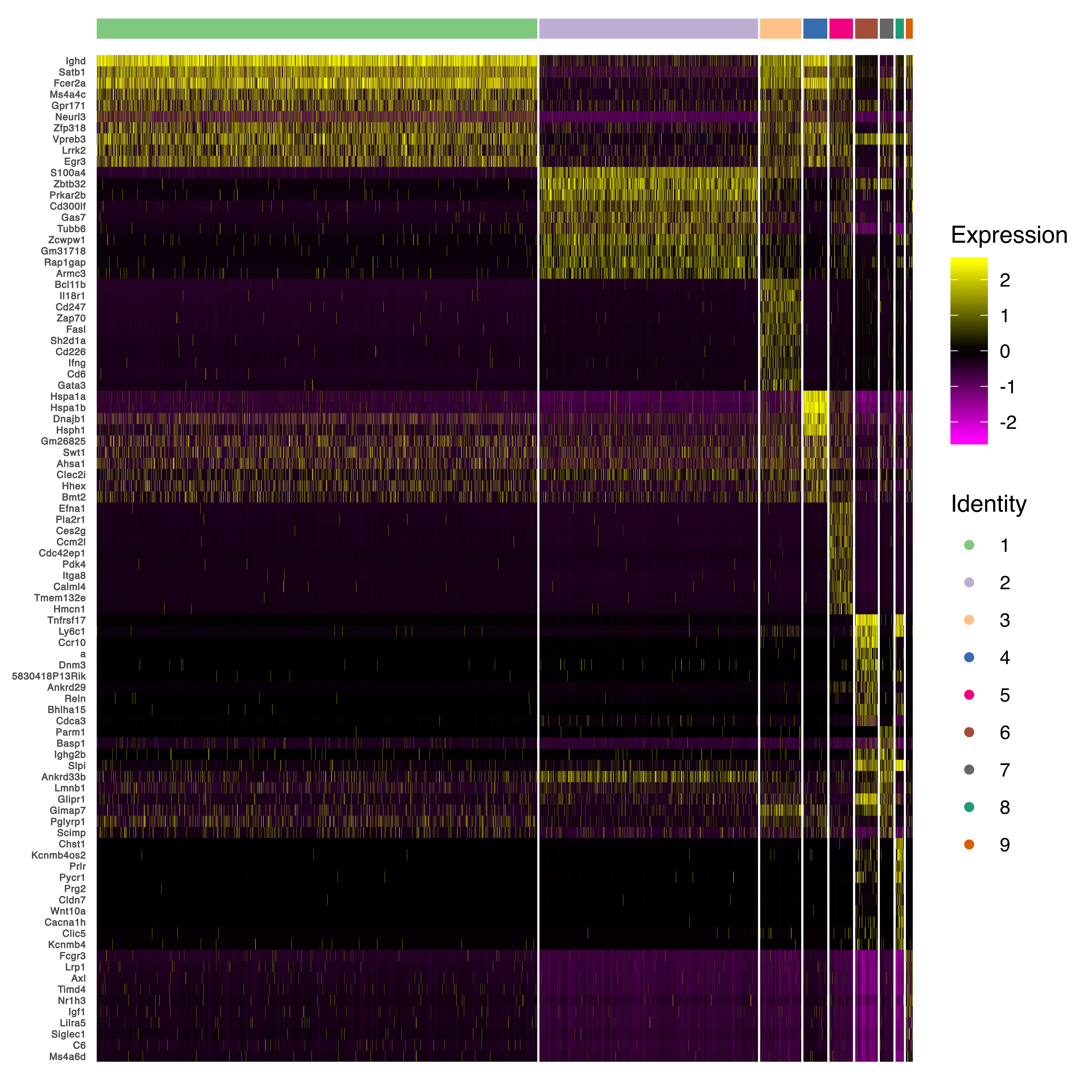

Supplement: Supplementary Figure 2 — | Heat map of the top 10 marker genes for the nine B cell clusters. The top 10 cluster-specific markers were selected based on the average log (fold change). [file Image_2.tif]
